# Qualitative description of work of a psychotherapy group in the context of COVID pandemic

**DOI:** 10.1192/j.eurpsy.2022.849

**Published:** 2022-09-01

**Authors:** I. Najbar, E. Guśtak, R. Młynarczyk, D. Łopalewska

**Affiliations:** 1 Babiński Clinical Hospital in Cracow, Center For Education, Research And Development, Karków, Poland; 2 Jagiellonian University Medical College, Katedra Psychiatrii Uj Cm, Zakład Terapii Rodzin I Psychosomatyki, Karków, Poland

**Keywords:** Psychosis, stationary psychiatric department, Psychotherapy, COVID pandemic

## Abstract

**Introduction:**

COVID pandemic very much influenced therapeutic organisation of psychiatric care. This also applies to our hospital. Especially therapeutic activities in stationary wards.

**Objectives:**

We would like to show changes occurring in psychotherapy group of the patients with psychotic disorder in the stationary ward in core of pandemic.

**Methods:**

I would like to present a qualitative description of psychotherapy group in the context of COVID. The group is designed for patients with experience of psychosis, grounded in psychodynamic school and has a long tradition at an acute admission in stationary psychiatric department.

**Results:**

During the pandemic there were epidemiological constraints. From six members of personnel in the basic assumptions, we reduced to minimum. So from two co-therapists, reflecting team and an observer, we ended leading the group every second time with one of the therapists. Despite of our efforts to maintain a continuous group, the group was closed for more than half a year and then reactivated based on old rules and roots, but less consistent memory of group members. During this most strict reduction of personnel, which would never have been accepted apart from the pandemic restrictions appeared a few interesting phenomenon. One of them was - twin groups. With the colleague we lead the group every second time. The group shows us a similar picture twice.

**Conclusions:**

As we understand, twin groups is a way to try to keep this group together in its already damaged setting. For the moment the abstract submission group is continuing to work within its present arrangement.

**Disclosure:**

No significant relationships.

